# Beyond Breathlessness Intensity: A Prospective Psychometric Validation of the Multidimensional Dyspnea Profile in Heart Failure with Reduced and Mildly Reduced Ejection Fraction

**DOI:** 10.3390/jcm15093533

**Published:** 2026-05-05

**Authors:** Monira I. Aldhahi, Rakan I. Nazer, Ali M. Albarrati

**Affiliations:** 1 Department of Rehabilitation Sciences, College of Health and Rehabilitation Sciences, Princess Nourah bint Abdulrahman University, Riyadh 11671, Saudi Arabia; mialdhahi@pnu.edu.sa; 2 Department of Cardiac Sciences, College of Medicine, King Saud University, Riyadh 11461, Saudi Arabia; raknazer@ksu.edu.sa; 3 Department of Rehabilitation Sciences, College of Applied Medical Sciences, King Saud University, Riyadh 11451, Saudi Arabia

**Keywords:** multidimensional dyspnea profile, heart failure with reduced ejection fraction, heart failure with mildly reduced ejection fraction, psychometric validation, COSMIN, confirmatory factor analysis, minimal clinically important difference

## Abstract

**Background/Objectives:** Dyspnoea in heart failure with reduced or mildly reduced ejection fraction (HFrEF/HFmrEF) is multidimensional, yet conventional unidimensional scales do not capture its sensory and affective components. The Multidimensional Dyspnea Profile (MDP) addresses this gap; however, its psychometric properties have not been established in a dedicated HFrEF/HFmrEF cohort. We assessed structural validity, internal consistency, test–retest reliability, and construct validity of the MDP using COSMIN methodology. **Methods:** In this prospective, single-centre psychometric validation study, 101 clinically stable adults with HFrEF or HFmrEF were enrolled at a tertiary outpatient cardiac clinic in Riyadh, Saudi Arabia. Participants completed the MDP alongside Dyspnea-12, modified Medical Research Council scale, Kansas City Cardiomyopathy Questionnaire-12, Fatigue Severity Scale, and 6 min walk test. Test–retest data were obtained at 12 days in patients confirmed stable by the Global Rating of Change (n = 87). Psychometric evaluation included Cronbach’s α, intraclass correlation (ICC_2_,_1_), standard error of measurement, minimum detectable change (MDC_95_), confirmatory factor analysis (comparative fit index [CFI], root mean square error of approximation [RMSEA], standardised root mean square residual [SRMR]), and 12 a priori construct hypotheses. A preliminary minimal clinically important difference (MCID) was estimated using anchor- and distribution-based methods. **Results:** The mean age was 55 ± 11 years and 80% were male. CFA supported the two-factor model (CFI = 0.96; RMSEA = 0.061; SRMR = 0.058). Cronbach α was 0.92 for the full scale, 0.88 for immediate perception, and 0.91 for emotional response. ICC_2,1_ was 0.94 (95% CI: 0.91–0.96), and MDC_95_ was 4.2 points. All 12 hypotheses were confirmed. The preliminary MCID was 8 points. **Conclusions:** The MDP is a reliable, valid, and clinically interpretable multidimensional dyspnoea measure in HFrEF/HFmrEF. The 8-point MCID is preliminary and requires confirmation in larger longitudinal intervention studies.

## 1. Introduction

Heart failure (HF) is a global pandemic affecting approximately 64 million people worldwide. HF with reduced ejection fraction (HFrEF) and HF with mildly reduced ejection fraction (HFmrEF) account for roughly half to two-thirds of cases in contemporary registries [1,2]. These conditions are characterised by progressive symptoms, impaired functional capacity, and high rates of hospitalisation and mortality [3,4]. Dyspnoea, defined as the subjective sensation of breathing difficulty, is the most prevalent and distressing symptom reported by patients with HFrEF/HFmrEF, and is a major driver of emergency presentations, hospital readmissions, and impaired quality of life [5]. Despite its central clinical importance, dyspnoea in heart failure remains incompletely measured in both clinical practice and research, largely because the most widely used instruments are unidimensional.

The New York Heart Association (NYHA) functional classification, and the modified Medical Research Council (mMRC) dyspnoea scale are commonly used instruments in heart failure practice. Both are ordinal scales that quantify the degree to which breathlessness limits physical activity, but neither captures the qualitative nature of the dyspnoea sensation nor its emotional and psychological consequences [6]. The Borg scale, though practical for exertional assessment, similarly provides only a single intensity rating without distinguishing sensory descriptors. This unidimensional approach is clinically limiting: two patients with identical NYHA class III ratings may have profoundly different dyspnoea experiences—one dominated by air hunger and suffocation, and the other by inspiratory effort and anxiety—with distinct physiological substrates and may respond differently to treatment [5,6,7,8].

Functional neuroimaging has established the neurobiological basis for dyspnoea’s multidimensional structure [9]. It has revealed that the affective component of breathlessness activates limbic and insular cortex regions shared with pain processing, whereas the sensory-discriminative component engages the somatosensory cortex. Consistent with this, the 2012 official American Thoracic Society statement characterises dyspnoea as having at least three distinct experiential domains—air hunger, work/effort of breathing, and chest tightness—each with distinct perceptual and neuroanatomical features [10]. This multidimensionality is particularly relevant in HFrEF, where elevated pulmonary venous pressure, reduced cardiac output, skeletal muscle deconditioning, and heightened sympathoadrenal activity collectively produce overlapping sensory and affective signals [11].

The Multidimensional Dyspnea Profile (MDP), developed by Banzett and colleagues [12], was designed to operationalise this neurobiological framework in a clinically deployable instrument. Specifically, the MDP quantifies breathing unpleasantness (A1), the intensities of five standardised sensory quality (SQ) descriptors, and five emotional responses (A2 domain: anxiety, frustration, depression, anger, and fright) using 0–10 numerical rating scales, yielding an immediate perception (A1 + SQ) subscale score (range 0–60), an emotional response subscale score (range 0–50), and a total score (range 0–110). The MDP has demonstrated strong psychometric properties in laboratory settings [12], emergency department patients [13], and mixed cardiorespiratory outpatient cohorts [14,15]. A systematic review conducted by Williams et al. [16] confirmed acceptable-to-excellent reliability and validity across various settings. However, the review highlighted that primary cardiac populations, particularly HFrEF/HFmrEF, were under-represented or entirely absent from published validation studies.

This evidence gap is consequential. The dyspnoea phenotype in HFrEF differs from that in chronic obstructive pulmonary disease (COPD) and interstitial lung disease in several important respects. It is more strongly modulated by pulmonary congestion and fluid redistribution, more closely linked to affective states such as anxiety and depression, and more responsive to haemodynamic interventions than to bronchodilators or oxygen [11,17]. Psychometric validation in COPD or mixed populations does not assure equivalent performance in HFrEF; floor and ceiling distributions, factor structure, and the relative salience of sensory versus affective domains may differ. Therefore, establishing COSMIN-compliant evidence for MDP measurement properties specifically in HFrEF/HFmrEF is a prerequisite for its use in clinical trials and guideline-recommended symptom monitoring. Importantly, dyspnoea in HFrEF/HFmrEF is not merely a psychometric construct—it is a primary therapeutic target and a clinically actionable symptom. Even after sequenced initiation and titration of the four pillars of guideline-directed medical therapy, a clinically meaningful proportion of patients continue to report persistent exertional dyspnoea. For these patients, the soluble guanylate cyclase stimulator, vericiguat is now positioned as an evidence-based add-on option in international position statements [18]. Adherence-orientated strategies, such as the polypill approach, are similarly relevant to closing the gap between guideline recommendations and real-world residual symptom burden [19]. A multidimensional dyspnoea instrument that distinguishes the sensory and affective components of residual symptoms is therefore not only a measurement tool but a clinically actionable adjunct to therapy optimisation in symptomatic HFrEF/HFmrEF.

This study therefore aimed to conduct a comprehensive psychometric evaluation of the MDP in a dedicated HFrEF/HFmrEF cohort, addressing: (1) structural validity via confirmatory factor analysis (CFA) of the two-factor model; (2) internal consistency; (3) test–retest reliability with clinical stability verification and anchor-based MCID estimation; and (4) construct validity against a priori convergent and divergent hypotheses using five concurrent outcome measures. We hypothesised that the MDP would demonstrate adequate structural validity and excellent reliability, and would show stronger correlations with dyspnoea-specific measures than with a divergent measure of fatigue.

## 2. Materials and Methods

### 2.1. Study Design, Setting, and Participants

This was a prospective psychometric validation study conducted at the specialised outpatient heart failure clinic of a tertiary cardiac referral centre in Riyadh, Saudi Arabia. We collected data consecutively from March to December 2023. Two trained research clinicians conducted all assessments during scheduled outpatient clinic visits, following standardised protocols. We conducted all procedures in accordance with the Declaration of Helsinki and obtained written informed consent from all participants before enrolment.

Eligible participants were adults aged ≥18 years with a physician-confirmed diagnosis of HFrEF (LVEF <40%) or HFmrEF (LVEF 40–49%), consistent with the 2021 European Society of Cardiology Heart Failure Guidelines [4]. Participants needed to be clinical stable, defined as no unplanned hospitalisation and no change in heart failure pharmacotherapy in the 30 days preceding enrolment. Participants were excluded if they had a concurrent diagnosis of known pulmonary disease (COPD, asthma, interstitial lung disease, or pulmonary arterial hypertension), an acute decompensated heart failure event in the preceding 30 days, cognitive impairment, inability to complete self-reported questionnaires, or inability to provide informed consent. Heart failure with preserved ejection fraction (HFpEF; LVEF ≥50%) was not included in this study, as the dyspnoea mechanisms and haemodynamic drivers in HFpEF differ substantively from those in HFrEF/HFmrEF.

### 2.2. Sample Size

Sample size was estimated following COSMIN statistical guidance [20]. For ICC estimation with a target lower bound of the 95% CI ≥ 0.80 (null ICC_0_ = 0.70, expected ICC_1_ = 0.90, α = 0.05, β = 0.20, two repeated measurements), a minimum of 51 participants is required for the reliability subsample. For construct validity, we assume Pearson r ≥0.40 requires n = 46 for 80% power (two-tailed α = 0.05). For CFA with six to eleven indicators per factor, a minimum of 100 participants is generally recommended [21]. Accordingly, we targeted n = 100, achieved n = 101 enrolled, with n = 87 patients confirmed stable for the reliability subsample.

### 2.3. Clinical and Functional Assessments

Demographic and clinical data, including age, sex, body mass index (BMI), LVEF, NYHA functional class (assigned by a heart failure specialist at the clinic visit), comorbidities, and current medications (including the four pillars of guideline-directed medical therapy, and loop diuretics), were extracted from the electronic health record and confirmed by structured interview. Resting heart rate, blood pressure, and peripheral oxygen saturation (SpO_2_) were measured at the beginning of the assessment session.

### 2.4. Patient-Reported Outcome Measures

#### 2.4.1. Multidimensional Dyspnea Profile (MDP)

The MDP is an 11-item instrument quantifying the sensory and affective dimensions of dyspnoea [12]. Breathing unpleasantness (A1) is rated on a 0 (“neutral”) to 10 (“unbearable”) scale. The intensities of five sensory quality (SQ) descriptors—air hunger, work/effort, chest tightness, mental effort, and a user-nominated sensation—are each rated 0 (“none”) to 10 (“as intense as I can imagine”). Five emotional responses (A2: anxiety, frustration, depression, anger, fright) are similarly rated 0–10. The immediate perception subscale (A1 + SQ: range 0–60) and emotional response subscale (A2: range 0–50) together constitute the total score (range 0–110), with higher scores indicating greater dyspnoea severity [22].

#### 2.4.2. Dyspnea-12 (D-12)

The D-12 is a 12-item multidimensional breathlessness questionnaire with two subscales: seven physical items describing sensory qualities, and five affective items capturing the emotional impact of dyspnoea [16,23]. Each item is scored from 0 (“none”) to 3 (“severe”), producing subscale and total scores (range 0–36).

#### 2.4.3. Modified Medical Research Council (mMRC) Dyspnea Scale

The mMRC is a five-grade ordinal scale assessing functional breathlessness from grade 0 (“not troubled by breathlessness except with strenuous exercise”) to grade 4 (“too breathless to leave the house, or breathless when dressing or undressing”) [6].

#### 2.4.4. Kansas City Cardiomyopathy Questionnaire-12 (KCCQ-12)

The KCCQ-12 is a 12-item disease-specific health status instrument for heart failure, generating domain scores (physical limitation, symptom frequency/severity, quality of life, social limitation) and an overall summary score (OSS) on a 0–100 scale, where higher scores reflect better health status [24,25].

#### 2.4.5. Fatigue Severity Scale (FSS)

The FSS is a nine-item scale assessing the impact of fatigue on daily functioning, with items rated on a 7-point Likert scale (1–7) and averaged to produce a total score [26,27,28]. Fatigue and dyspnoea are related but conceptually distinct symptoms in HFrEF with partially separable physiological substrates [17].

#### 2.4.6. Six-Minute Walk Test (6MWT)

Functional exercise capacity was assessed using the 6MWT according to American Thoracic Society guidelines [29], conducted in a flat indoor corridor of 30 m. Six-minute walk distance (6MWD, metres) was recorded.

#### 2.4.7. Global Rating of Change (GRC) Scale

The GRC was administered at the retest visit (12 days after baseline) to classify each participant’s perceived change in dyspnoea as improved (GRC +2 to +5), stable (GRC −1 to +1), or worsened (GRC −5 to −2) [29].

### 2.5. Data Analysis

All analyses were performed in IBM SPSS Statistics Version 30.0 (Armonk, NY, USA) and R version 4.3.2 (lavaan package v.0.6-17 for CFA). Statistical significance was set at α = 0.05 (two-tailed). Continuous variables are presented as mean ± standard deviation (SD) or median (interquartile range, IQR) based on the result of the Shapiro–Wilk normality test; categorical variables are presented as frequency (percentage).

#### 2.5.1. Floor and Ceiling Effects

Floor and ceiling effects were examined for the MDP total score and each subscale. A floor or ceiling effect was considered present if >15% of participants scored the minimum or maximum possible value, respectively [30].

#### 2.5.2. Structural Validity

The hypothesised two-factor structure of the MDP (Factor 1: immediate perception subscale comprising A1 and five SQ items; Factor 2: emotional response subscale comprising five A2 items) was evaluated using CFA with maximum likelihood estimation. Model fit was assessed using the following indices: comparative fit index (CFI; acceptable ≥0.90, good ≥0.95), root mean square error of approximation (RMSEA; acceptable ≤0.08, good ≤0.06), standardised root mean square residual (SRMR; acceptable ≤0.10, good ≤0.08), and Tucker–Lewis Index (TLI; acceptable ≥0.90) [31]. Factor loadings, inter-factor correlation, and modification indices were inspected.

#### 2.5.3. Reliability Analysis

Internal consistency was evaluated using Cronbach’s alpha (α) for the full MDP scale and each subscale independently. Values ≥ 0.90 are considered excellent [30]. Test–retest reliability was assessed using the two-way mixed-effects, single-measures ICC (ICC_2,1_) model in the clinically stable subsample (GRC −1 to +1; n = 87). ICC values ≥ 0.90 represent excellent reliability; 0.75–0.89 good reliability; 0.50–0.74 moderate reliability; <0.50 poor reliability [32]. The standard error of measurement (SEM) was computed as SEM = SD × √(1 − ICC). The minimum detectable change at the 95% confidence level was calculated as MDC_95_ = SEM × 1.96 × √2. Bland–Altman analysis was used to assess for proportional bias and systematic error [33].

#### 2.5.4. Anchor-Based Minimal Clinically Important Difference (MCID)

We estimated an anchor-based MCID using the GRC scale as the external anchor. We calculated the mean change in MDP among stable participants (GRC −1 to +1) and among those reporting slight improvement (GRC +2) to establish the MCID threshold. We performed receiver operating characteristic (ROC) curve analysis to identify the optimal MDP change score for discriminating slight improvement (GRC +2) from no change (GRC 0/±1) by maximising the Youden index. This estimate was interpreted alongside the distribution-based MDC_95_.

#### 2.5.5. Construct Validity

Construct validity was evaluated by specifying twelve directional hypotheses before data analysis, following COSMIN recommendations [20]. Hypotheses specified the expected direction (positive or negative) and magnitude (weak <0.40; moderate 0.40–0.59; strong 0.60–0.79; very strong ≥0.80) of each correlation. Pearson correlation (r) was used for normally distributed continuous comparators, and Spearman rank-order correlation (*rs*) was used for ordinal scales (mMRC) and non-normally distributed variables. COSMIN requires confirmation of ≥75% of pre-specified hypotheses for a verdict of sufficient construct validity. Both total-scale and subscale-level analyses were performed to evaluate whether the MDP sensory and affective subscales showed theoretically predicted differential associations with functional versus emotional comparator measures (subscale specificity hypothesis) (Table 1).

#### 2.5.6. Distribution-Anchored Severity Band Derivation

To support clinical interpretability of MDP scores in the absence of established external normative data for HFrEF/HFmrEF populations, distribution-anchored severity bands were derived using the mean ± 1 SD approach, a distributional method commonly used in patient-reported outcome instrument development and reference range derivation [30].

## 3. Results

### 3.1. Participant Characteristics

**Figure 1:**
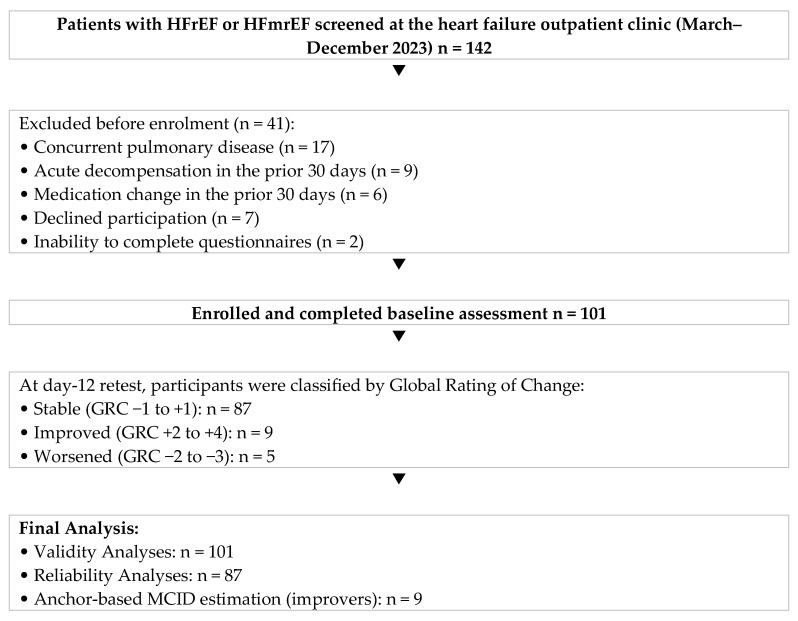
Study flow diagram. HFmrEF: heart failure with mildly reduced ejection fraction. HFrEF: heart failure with reduced ejection fraction. GRC: Global Rating of Change. MCID: minimal clinically important difference.

A total of 101 adults with HFrEF or HFmrEF were enrolled (Figure 1). Of these, 87 (86%) met the clinical stability criterion (GRC −1 to +1) at the 12-day retest visit and were included in the reliability analyses. Fourteen participants (14%) were classified as changed (n = 9 improved [GRC +2 to +4]; n = 5 worsened [GRC −2 to −3]) and were excluded from the reliability subsample but were retained in all cross-sectional analyses.

The mean age was 55 ± 11 years, and 80% were male. NYHA classification was class II in 57 (57%) and class III in 44 (43%) participants. Mean LVEF was 35 ± 15%; 62 participants (61%) had HFrEF (LVEF < 40%) and 39 (39%) had HFmrEF (LVEF 40–49%). The most prevalent comorbidities and medication are presented in Table 2. No patients were receiving oral or inhaled corticosteroid therapy.

### 3.2. Floor and Ceiling Effects

Three participants (3.0%) scored the minimum MDP total score (0) and ten participants (9.9%) scored the maximum (110). Neither value exceeded the pre-specified 15% threshold; therefore, no significant floor or ceiling effects were observed. Subscale analysis revealed similarly acceptable distributions: the IP subscale showed 2.0% floor and 8.9% ceiling effects, and the ER subscale showed 4.0% floor and 7.9% ceiling effects.

### 3.3. Structural Validity

CFA of the hypothesised two-factor model demonstrated acceptable-to-good fit: CFI = 0.96, TLI = 0.95, RMSEA = 0.061 (90% CI: 0.041–0.079), SRMR = 0.058. All factor loadings were statistically significant (*p* < 0.001) and ranged from 0.61 to 0.89 for the IP factor and from 0.72 to 0.91 for the ER factor, indicating strong item–factor relationships within each subscale. The inter-factor correlation was r = 0.74, confirming that the two domains are related but empirically distinct, consistent with their theoretical independence.

No modification indices exceeded 10.0 in a way that would suggest theoretically meaningful cross-loadings. A single-factor (unidimensional) model was also tested as a comparison; it demonstrated substantially poorer fit (CFI = 0.81, RMSEA = 0.118, SRMR = 0.091), confirming the superiority of the two-factor structure and the empirical distinctness of the sensory and affective dyspnoea dimensions in this population.

### 3.4. Reliability and Measurement Error

Internal consistency was excellent across all levels: full scale α = 0.92, IP subscale α = 0.88, ER subscale α = 0.91. Test–retest reliability in the 87 clinically stable participants was excellent: ICC_2,1_ = 0.94 (95% CI: 0.91–0.96). The SEM was 1.5 points and the MDC_95_ was 4.2 points, indicating that a total MDP score change of ≥4.2 points represent genuine change beyond measurement error. Bland–Altman analysis demonstrated narrow limits of agreement (mean difference = −0.78 points; 95% limits: −6.33 to +3.77) with no evidence of systematic bias or proportional error (Pearson r between mean scores and difference scores: r = 0.08, *p* = 0.45). These findings are summarised in Table 3.

### 3.5. Anchor-Based MCID Estimation

Of the 14 participants classified as changed at retest, nine reported slight improvement (GRC +2) and five reported worsening (GRC −2 to −3). Among the nine improvers, mean MDP total score decreased by 7.8 ± 3.2 points from baseline to retest. ROC analysis identified an MDP change score of −8 points as the optimal cut-point for discriminating slight improvement (GRC +2) from no change (GRC −1 to +1), with area under the curve (AUC) = 0.84 (95% CI: 0.73–0.95), sensitivity = 0.78, and specificity = 0.81. Accordingly, an anchor-based MCID of 8 points is therefore proposed as a preliminary estimate.

### 3.6. Construct Validity

All 12 a priori hypotheses (100%) were confirmed, exceeding the COSMIN ≥75% threshold for sufficient construct validity. The total MDP score correlated strongly and positively with D-12 total score (r = 0.72, *p* < 0.001) and moderately-to-strongly with mMRC (ρ = 0.67, *p* < 0.001), supporting convergent validity with dyspnoea-specific comparators. The MDP correlated strongly and negatively with KCCQ-12 overall summary score (r = −0.70, *p* < 0.001) and moderately with 6MWD (r = −0.65, *p* < 0.001), consistent with the expected relationship between dyspnoea burden and health status/function. The correlation with FSS (r = 0.57, *p* < 0.001) supported the discriminant hypothesis that MDP does not simply measure fatigue.

Subscale-level analyses confirmed the hypothesised specificity of MDP domains. The MDP IP subscale correlated more strongly with D-12 physical subscale (r = 0.70) than with D-12 affective subscale (r = 0.55), and more strongly with mMRC (ρ = 0.64) and 6MWD (r = −0.67) than the ER subscale did (mMRC ρ = 0.54; 6MWD r = −0.48). Conversely, the MDP ER subscale correlated more strongly with D-12 affective subscale (r = 0.74) than with D-12 physical subscale (r = 0.52), and more strongly with KCCQ-12 social limitation domain (r = −0.62) and FSS (r = 0.64) than the IP subscale did (KCCQ-12 social: r = −0.44; FSS: r = 0.41). These cross-domain patterns indicate that the MDP’s sensory and affective subscales capture meaningfully distinct aspects of the dyspnoea experience in HFrEF/HFmrEF.

### 3.7. Distribution-Anchored Severity Bands and Subscale Profile Analysis

To support the clinical interpretation of MDP scores, distribution-based severity bands were derived from the present cohort’s observed score distributions. Using the conventional mean ± 1 SD approach, three severity strata were defined for the MDP total score and for each subscale independently: mild (below mean − 1 SD), moderate (within mean ± 1 SD), and severe (above mean + 1 SD). Based on the observed distributions (total score: 56 ± 11; IP subscale: 30 ± 8; ER subscale: 26 ± 7), the resulting thresholds are presented in Table 4.

Subscale profile analysis revealed that the IP/ER ratio provides clinically discriminative information beyond the MDP total score. Patients with similar total scores showed divergent subscale configurations. A sensory-dominant profile, defined as IP > 38 with ER < 19, was associated with higher mMRC grades (*rs* = 0.64) and lower 6MWD (r = −0.67), whereas an affective-dominant profile, defined as ER > 33 with IP < 22, was associated with higher FSS scores (r = 0.64) and greater KCCQ-12 social limitation (r = −0.62).

## 4. Discussion

This study provides the first comprehensive COSMIN-guided psychometric validation of the MDP in a dedicated HFrEF/HFmrEF population. The principal findings were that the hypothesised two-factor sensory/affective structure was confirmed by CFA with acceptable-to-good model fit, internal consistency was excellent at both the scale and subscale levels, test–retest reliability was excellent in clinically stable patients, all 12 a priori construct-validity hypotheses were confirmed, and distribution-anchored severity bands identified clinically interpretable subgroups. Together, these findings support the MDP as a psychometrically robust and clinically relevant instrument for multidimensional dyspnoea assessment in HFrEF/HFmrEF.

The confirmation of the MDP’s two-factor structure in this cohort is an important advance beyond prior studies, which have generally reported internal consistency without formal factor analysis. The model fit indices and the markedly poorer fit of the unidimensional model indicate that dyspnoea in HFrEF/HFmrEF is not adequately represented by a single latent construct. This has direct implications for clinical practice because reliance on a total score alone may obscure the relative contribution of sensory and affective components that could inform management. For example, a patient with a disproportionately elevated ER subscale score may warrant greater attention to anxiety, depression, or distress-related symptom amplification, whereas a more sensory-dominant profile may reflect a predominantly haemodynamic or exertional burden.

The inter-factor correlation indicated that the sensory and affective domains are closely related but empirically distinct [14,15]. This is consistent with the expected overlap between the perception of breathlessness and the emotional response it evokes. Importantly, the subscale-specific validity analyses reinforced this distinction, as the IP and ER subscales showed different correlational patterns with functional, symptom-specific, and psychosocial comparators. These findings support the value of retaining the two subscales separately rather than collapsing them into a single summary score.

Reliability was excellent across the full scale and both subscales, and the test–retest ICC was high in the clinically stable subsample [13,16]. The use of GRC-anchored stability verification strengthens confidence in the reproducibility estimate because it reduces the risk of misclassifying true clinical change as measurement instability. The MDC95 of 4.2 points provides a useful benchmark for distinguishing signal from noise in individual patients. In practical terms, changes smaller than this threshold are unlikely to exceed measurement error, whereas larger changes are more likely to reflect real symptom change.

The anchor-based MCID of 8 points is a clinically useful but preliminary estimate. It is larger than the MDC95, which is expected because a change must exceed measurement error before it can be perceived as meaningful. The difference between these two values is important: the MDC95 defines the lower bound for a reliable change, whereas the MCID approximates the smallest change that patients are likely to perceive as important. Because the MCID was derived from a small subgroup of improvers, it should be interpreted cautiously and validated in larger longitudinal cohorts before being used as a firm clinical benchmark.

The confirmation of all 12 pre-specified construct-validity hypotheses, including four subscale specificity hypotheses, represents a substantially more rigorous validity assessment than the simple correlation matrices reported in prior MDP studies [13,14,15,16,22,34]. The pattern of results across convergent, divergent, and discriminant comparators is consistent with the theoretical framework of multidimensional dyspnoea and with the pathophysiology of HFrEF/HFmrEF. The strong correlation between MDP total score and KCCQ-12 confirms that dyspnoea severity is closely coupled with heart failure-specific health status. This finding extends the work of Kupper et al., who identified dyspnoea as the strongest determinant of health-related quality of life in a cohort of 271 patients with chronic HF [17].

The relationship between MDP total score and 6MWT is also consistent with the known role of exertional dyspnoea in limiting exercise performance in HFrEF [11]. The moderate correlation with FSS, while statistically significant, was weaker than those with dyspnoea-specific measures. This supports the discriminant hypothesis and indicates that the MDP primarily measures dyspnoea rather than the overlapping construct of fatigue. The subscale-level correlations added further interpretive value. The IP subscale showed stronger relationships with dyspnoea severity and exercise capacity, whereas the ER subscale showed stronger associations with affective burden, social limitation, and fatigue. This pattern is clinically plausible and suggests that the MDP captures more than symptom intensity alone. It also supports the view that the emotional burden of dyspnoea may contribute to broader health status impairment in a way that is not fully captured by functional scales [17].

The derivation of distribution-anchored severity bands from the present cohort’s observed score distributions represent a pragmatic first step toward operationalising the MDP for routine clinical use in HFrEF/HFmrEF. Using the mean ± 1 SD convention—a well-established distributional approach for generating preliminary interpretive reference ranges in the absence of external normative data—three severity strata (mild, moderate, severe) were defined for the MDP total score and for each subscale independently. In the present cohort of stable tertiary outpatients, the moderate range spans 45–67 points for the total score, 22–38 for the IP subscale, and 19–33 for the ER subscale, reflecting the distributional characteristics of a clinically stable, predominantly NYHA class II–III population on optimised guideline-directed medical therapy. These thresholds are explicitly exploratory: they characterise the symptom burden distribution within a specific clinical context and should not be treated as validated diagnostic cut-points until independently replicated in community-based, sex-balanced, and multicentre HFrEF/HFmrEF cohorts.

Nonetheless, the interpretive value of the severity bands is substantially enhanced when considered alongside the IP/ER subscale profile rather than the total score in isolation. The results of the present study demonstrate that patients with equivalent MDP total scores exhibit fundamentally divergent subscale configurations that correspond to distinct functional and psychosocial phenotypes Two patients with an identical total score may differ profoundly in clinical phenotype: one driven by predominantly sensory burden and the other by predominantly affective burden. The sensory dominant pattern should prompt reassessment of haemodynamic optimisation—diuretic adequacy, β-blocker tolerability, SGLT2 inhibitor uptake, sacubitril–valsartan titration, and consideration of vericiguat in patients with persistent symptoms despite guideline-directed medical therapy [18] together with structured cardiac rehabilitation. The affective-dominant pattern should prompt screening for anxiety, depression, and panic-related dyspnoea, with consideration of cognitive-behavioural therapy, breathing-control techniques, and where indicated, anxiolytic management. We emphasise that these bands are derived from a single tertiary cohort and are exploratory; they require external confirmation before being adopted as definitive cut-points.

The psychometric properties observed in this HFrEF/HFmrEF cohort align with those reported in prior MDP validation studies [12,13,14,22,34]. Internal consistency in the present study (α 0.88–0.92) matches the ranges reported by Meek et al. [13] and Ekström et al. [14]. Test–retest reliability (ICC 0.94) is at the upper end of the published range, consistent with our use of GRC-anchored stability verification. The convergent correlation with D-12 (r = 0.72) closely matches the Arabic chronic respiratory cohort of Shaheen et al. [22] and the Swedish cardiorespiratory cohort of Ekström et al. [14]. Importantly, the present study is the first to apply confirmatory rather than exploratory factor analysis to the MDP in a dedicated HFrEF/HFmrEF population, and to demonstrate explicit subscale specificity using twelve a priori construct-validity hypotheses.

Although the Japanese version of the MDP has been linguistically validated only in COPD outpatients to date [34], that work is mechanistically relevant to the HFrEF/HFmrEF setting. Kanezaki et al. demonstrated that the air-hunger descriptor of the MDP correlated significantly and inversely with total walking-derived energy expenditure (r = −0.47) and that the anxiety and depression items correlated inversely with both the amount and intensity of accelerometer-quantified physical activity (r = −0.49 and r = −0.46). This translation of multidimensional dyspnoea into objective physical activity behaviour is directly applicable to HFrEF/HFmrEF, in which physical inactivity is both a consequence of dyspnoea and a modifiable target of cardiac rehabilitation. The present study now provides the psychometric foundation for analogous accelerometer-paired analyses in HFrEF/HFmrEF, in which the MDP IP and ER subscales could be used as patient-reported anchors of dyspnoea-driven behavioural inactivity, complementing functional measures such as the 6MWT.

### 4.1. Clinical Implications

The MDP’s clinical utility may be embedded within the heart failure therapy-optimisation pathway. In a patient with a persistently high IP-dominated profile despite four-pillar guideline-directed medical therapy, the IP signal supports objective reassessment of congestion and consideration of vericiguat or further haemodynamic optimisation [18]. In a patient with a persistently high ER-dominated profile, the affective signal supports targeted screening for anxiety and depression, and structured psychological support. In patients with poor adherence-related residual symptoms, simplified regimens such as a polypill strategy may be useful [19]. Used in these ways, the MDP shifts dyspnoea measurement from a passive descriptor to an actionable component of contemporary heart failure care.

Furthermore, the MDP’s clinical utility can be amplified when paired with multiparametric ambulatory monitoring strategies, which are increasingly available in contemporary heart failure care. For instance, wearable cardioverter-defibrillators and other ambulatory devices offer continuous physiological signals—heart rate variability, thoracic impedance, activity counts, and arrhythmic events—that can detect transient clinical risk and worsening heart failure trajectories preceding overt decompensation [35,36]. These device-derived signals are objective but symptom-blind: a falling activity count and a small drop in 6 min walk distance may reflect genuine functional decline, but they cannot distinguish a sensory-dominant decompensation pattern from an affective-dominant pattern, nor identify the patient who is becoming more anxious about exertion despite preserved haemodynamics. The MDP, with its subscales and clinically interpretable severity bands, is well-suited to provide the patient-experience layer that contextualises device-derived data and enables targeted intervention.

The MDP can be used to structure cardiac rehabilitation programme and monitor cardiac rehabilitation-mediated change [37]. Exercise-based cardiac rehabilitation is a Class I recommendation in HFrEF and HFmrEF, reducing the risk of HF-related hospitalisation and improving health-related quality of life [4,38]. Despite this, dyspnoea is consistently the most cited reason for non-completion of exercise sessions and for premature discontinuation of rehabilitation. The MDP can support exercise prescription according to the IP/ER profile. Patients with a high IP-dominant profile may benefit from the addition of inspiratory muscle training and respiratory pattern coaching to the exercise prescription, whereas a high ER-dominant profile may benefit from the integration of cognitive-behavioural and breathing-control techniques addressing exertion-related anxiety.

### 4.2. Limitations

Several limitations must be acknowledged. First, this study was restricted to HFrEF and HFmrEF; results cannot be generalised to HFpEF, which now accounts for approximately half of all heart failure cases and is increasing in prevalence. The two-factor structural model is grounded in the neurobiology of dyspnoea processing and is, on theoretical grounds, expected to replicate in HFpEF. However, the relative magnitudes of the IP and ER subscales—and consequently the mean total score, the population-derived severity bands, and the MCID—are likely to differ. Dyspnoea in HFpEF is more frequently driven by exertion-induced rises in left atrial pressure, chronotropic incompetence, pulmonary hypertension, obesity, frailty, and high comorbid anxiety in older women, and may produce a different IP/ER profile and floor/ceiling distribution than in HFrEF/HFmrEF [39]. A dedicated HFpEF validation study with re-estimation of measurement properties, severity bands, and MCID is therefore a priority next step.

Second, the sample was predominantly male (80%), limiting generalisability to women with HFrEF/HFmrEF. Future studies should aim for sex-balanced enrolment to determine whether sensory descriptors and emotional reactivity to dyspnoea differ between males and females. Third, although this study was conducted at a single tertiary cardiac centre, this facility receives a diverse spectrum of heart failure cases from across the country.

Fourth, the MCID estimate is derived from a small number of improvers and should be regarded as preliminary, and confirmation in a prospective intervention trial is required. Fifth, 44% of participants had comorbid diabetes mellitus, which may independently affect symptom perception and emotional responses via autonomic neuropathy and mood-related mechanisms; whether diabetes modifies MDP performance warrants dedicated subgroup analysis in larger samples.

Sixth, although test–retest reliability was excellent during a 12-day period of verified clinical stability, the present design does not establish responsiveness within the COSMIN framework. Stability data quantify measurement error in the absence of true change; they do not demonstrate that the MDP can detect change after a therapeutic intervention. We did not evaluate within-patient change after structured cardiac rehabilitation, after up-titration of guideline-directed medical therapy, after initiation of vericiguat or SGLT2 inhibitors in patients with persistent dyspnoea despite optimised therapy, or after rehabilitation-mediated improvements in 6 min walk distance—settings in which dyspnoea relief is a primary therapeutic target [38]. Adequately powered longitudinal studies, with a priori responsiveness hypotheses on the expected direction and magnitude of change-score correlations and known-groups change effects, are needed to establish responsiveness and to confirm the MCID for the IP and ER subscales separately.

## 5. Conclusions

The MDP demonstrated excellent structural validity, internal consistency, test–retest reliability, and construct validity in adults with HFrEF/HFmrEF. The instrument provides a validated, multidimensional, and practically deployable tool that captures aspects of the dyspnoea experience in HFrEF/HFmrEF that are not captured by conventional unidimensional scales. The MDP phenotyping may be clinically meaningful because it supports more tailored clinical management, including optimisation of guideline-directed medical therapy, consideration of add-on therapy in patients with persistent symptoms, and structured cardiac rehabilitation. The 8-point change derived in this cohort is a preliminary anchor-based MCID estimate generated from a small subgroup of nine improvers and should be interpreted cautiously; the more conservative MDC95 of 4.2 points is recommended as the interim threshold for individual-patient interpretation. These estimates, along with the proposed severity bands, should be applied cautiously pending validation in more diverse settings.

## Figures and Tables

**Table 1:** A priori hypotheses for construct validity.

Comparator	Type	Direction	Expected Magnitude	Rationale
D-12 total	Convergent	Positive	Strong (r ≥ 0.60)	Both measure multidimensional dyspnoea severity.
D-12 physical subscale	Convergent	Positive	Strong (r ≥ 0.60)	Shared sensory content with MDP IP subscale.
D-12 affective subscale	Convergent	Positive	Strong (r ≥ 0.60)	Shared affective content with MDP ER subscale.
mMRC	Convergent	Positive	Moderate–strong (r ≥ 0.50)	Both measure dyspnoea; mMRC limited to functional domain.
KCCQ-12 overall summary	Convergent	Negative	Moderate–strong (r ≥ 0.50)	Greater dyspnoea → worse health status.
KCCQ-12 social limitation	Divergent	Negative	Weaker than D-12 (r 0.30–0.50)	Social domain less proximate to dyspnoea sensation.
6MWT distance	Convergent	Negative	Moderate (r ≥ 0.40)	More dyspnoea → worse exercise performance.
FSS total	Discriminant	Positive	Weaker than D-12 (r <0.60)	Related but conceptually distinct symptom.
MDP IP vs. D-12 physical	Subscale specificity	Positive	Stronger than IP vs. D-12 affective	Sensory content convergence.
MDP ER vs. D-12 affective	Subscale specificity	Positive	Stronger than ER vs. D-12 physical	Affective content convergence.
MDP ER vs. KCCQ-12 social	Subscale specificity	Negative	Stronger than IP vs. KCCQ-12 social	Emotional burden → social impact.
MDP IP vs. 6MWT	Subscale specificity	Negative	Stronger than ER vs. 6MWT	Sensory burden drives exercise limitation more directly.

Abbreviation: MDP = Multidimensional Dyspnea Profile; IP = immediate perception subscale; ER = emotional response subscale; D-12 = Dyspnea-12; mMRC = modified Medical Research Council; KCCQ-12 = Kansas City Cardiomyopathy Questionnaire-12; 6MWT = Six-Minute Walk Test; FSS = Fatigue Severity Scale.

**Table 2:** Demographic, clinical, pharmacological and outcome characteristics of participants (N = 101).

Variable	Mean ± SD or n (%)
**Age, years**	55 ± 11
**Sex, male**	81 (80%)
**BMI, kg/m^2^**	31.4 ± 4.5
**Education level—Elementary**	5 (5%)
**Education level—High school**	60 (59%)
**Education level—Bachelor’s degree**	33 (33%)
**Education level—Postgraduate**	3 (3%)
**HFrEF (LVEF < 40%)**	62 (61%)
**HFmrEF (LVEF 40–49%)**	39 (39%)
**LVEF, %**	35 ± 15
**NYHA class II/III**	57 (57%)/44 (43%)
**Hypertension**	78 (77%)
**Diabetes mellitus**	44 (44%)
**Hyperlipidaemia**	27 (27%)
**Beta-blocker**	96 (95%)
**ACE inhibitor/ARB/ARNI**	94 (93%)
**Mineralocorticoid receptor antagonist**	78 (77%)
**SGLT2 inhibitor**	71 (70%)
**Loop diuretic**	82 (81%)
**MDP total score**	56 ± 11
**IP subscale**	30 ± 8
**ER subscale**	26 ± 7
**Dyspnea-12 total**	26 ± 8
**KCCQ-12 overall summary score**	76.9 ± 22.3
**FSS total**	3.96 ± 1.91
**6MWT, metres**	315 ± 90

Abbreviation: ACE = angiotensin-converting enzyme; ARB = angiotensin II receptor blocker; ARNI = angiotensin receptor–neprilysin inhibitor; BMI = body mass index; ER = emotional response; FSS = Fatigue Severity Scale; HFmrEF = heart failure with mildly reduced ejection fraction; HFrEF = heart failure with reduced ejection fraction; IP = immediate perception; KCCQ-12 = Kansas City Cardiomyopathy Questionnaire-12; LVEF = left ventricular ejection fraction; MDP = Multidimensional Dyspnea Profile; NYHA = New York Heart Association; SGLT2 = sodium–glucose cotransporter-2; 6MWT = Six-Minute Walk Test.

**Table 3:** Reliability and measurement error statistics for the MDP.

Parameter	Full Scale	IP Subscale	ER Subscale	Interpretation
**Cronbach’s alpha (α)**	0.92	0.88	0.91	Excellent (≥0.90)
**ICC_2_,_1_ (95% CI)**	0.94 (0.91–0.96)	0.91 (0.88–0.94)	0.93 (0.90–0.95)	Excellent (≥0.90)
**SEM (points)**	1.5	1.2	1.1	Small relative to scale range
**MDC_95_ (points)**	4.2	3.3	3.1	Threshold for genuine change
**Bland–Altman mean difference**	−0.78	−0.41	−0.37	No systematic bias
**95% Limits of Agreement**	−6.33 to +3.77	−4.21 to +3.39	−3.98 to +3.24	Narrow; clinically acceptable

Abbreviation: ER = emotional response subscale; GRC = Global Rating of Change; ICC = intraclass correlation coefficient; IP = immediate perception subscale; MCID = minimal clinically important difference; MDC_95_ = minimum detectable change at 95% confidence; SEM = standard error of measurement.

**Table 4:** Distribution-anchored severity bands for clinical interpretation of MDP scale and subscales in stable HFrEF/HFmrEF participants (N = 101).

Score	Mild (<Mean − 1 SD)	Moderate (Mean ± 1 SD)	Severe (>Mean + 1 SD)
MDP total (0–110)	<45	45–67	>67
IP subscale (0–60)	<22	22–38	>38
ER subscale (0–50)	<19	19–33	>33

Abbreviations: ER = emotional response subscale; IP = immediate perception subscale; MDP = Multidimensional Dyspnea Profile.

## Data Availability

De-identified data supporting the findings of this study are available from the corresponding author upon reasonable request and after confirmation that data use complies with institutional and ethical regulations.
